# ﻿Diversity of flesh flies (Sarcophagidae, Sarcophaginae) of pond habitats in rural areas in the Croatian part of Baranja

**DOI:** 10.3897/zookeys.1159.100878

**Published:** 2023-04-24

**Authors:** Stjepan Krčmar

**Affiliations:** 1 Department of Biology, Josip Juraj Strossmayer University of Osijek, Cara Hadrijana 8/A, HR-31000 Osijek, Croatia Josip Juraj Strossmayer University of Osijek Osijek Croatia

**Keywords:** Baranja, Croatia, Diptera, flesh flies, Sarcophagidae, Sarcophaginae

## Abstract

The diversity of grey flesh flies (Sarcophagidae: Sarcophaginae) from the Croatian part of Baranja was studied during 2019 to 2021, resulting in 37 species, of which the following are new for the area: *Raviniapernix* (Harris, 1780); Sarcophaga (Het.) depressifrons Zetterstedt, 1845; S. (Het.) filia Rondani, 1860; S. (Het.) haemorrhoides Böttcher, 1913; S. (Het.) pumila Meigen, 1826; S. (Het.) vagans Meigen, 1826; S. (Lis.) dux Thomson, 1869; S. (Lis.) tuberosa Pandellé, 1896; S. (Meh.) sexpunctata (Fabricius, 1805); S. (Pan.) protuberans Pandellé, 1896; S. (Sar.) carnaria (Linnaeus, 1758); S. (Sar.) variegata (Scopoli, 1763), and S. (Pse.) spinosa Villeneuve, 1912. New locality records are provided for 25 species. Sarcophaga (Sar.) croatica Baranov, 1941 was the most abundant with 37%, followed by S. (Sar.) lehmanni Müller, 1922 (21%), and S. (Pas.) albiceps Meigen, 1826 (5%), making up 63% of all collected specimens. Most species (35) were collected in locality of Zmajevac, while the fewest (3) were collected in Bilje locality. During this study, S. (Pse.) spinosa was recorded in Croatia for the first time. Combined with previous records, 42 species of flesh flies have been recorded from Croatian Baranja, which comprise 27% of the flesh flies known to occur in Croatia. The total number of species of the family Sarcophagidae currently known in Croatia has increased to 156.

## ﻿Introduction

The European fauna of flesh flies (Sarcophagidae) comprises 310 species (Pape et al. 2015; Whitmore et al. 2020), of which approximately 150 species occur in Central Europe (Povolný and Verves 1997) and 155 species have been recorded from Croatia (Krčmar et al. 2019; Verves and Barták 2021). Verves and Barták (2021) erroneously listed *Paragusiamultipunctata* (Rondani, 1859) as new for Croatia, but this species was already listed by Krčmar et al. (2019) under the name *Taxigrammamultipunctata* (Rondani, 1859). Also, under a broad concept of *Sarcophaga*, the species name Heteronychia(s.str.)rohdendorfi (Povolný & Slamečkova, 1959) represents a junior secondary homonym of *Sarcophagarohdendorfi* Salem, 1936, *Parasarcophagarohdendorfi* Baranov, 1938, and *Sarcophagarohdendorfi* Baranov, 1941 (Whitmore 2011), and the valid name for this species is Sarcophaga (Heteronychia) lederbergi (Lehrer, 1995). Of the Croatian Sarcophagidae, most species (108) belong to subfamily Sarcophaginae, followed by Miltogramminae (38) and Paramacronychiinae (9) (Krčmar et al. 2019; Verves and Barták 2021). By reviewing the published data of Langhoffer (1920), Baranov (1928, 1929, 1938, 1941, 1942, 1943), Strukan (1964, 1967, 1970), Sisojević et al. (1989), Rucner (1994), as well as the recently published data of Whitmore (2010, 2011), Whitmore et al. (2013), and Krčmar et al. (2019), 90 species of flesh flies have been recorded in the Pannonian–Peripannonian biogeographic region of Croatia (Sarcophaginae: 68; Miltogramminae: 19; Paramacronychiinae: 3). Most of the studies were done and most species were recorded in the northern part of this region. In the recent study of Krčmar et al. (2019), 29 species of flesh flies were collected from only two localities in the Croatian part of Baranja, which is far from the total number of species considering the great landscape diversity of this area. The principal aims of this study are to give the first overview of the flesh fly fauna from subfamily Sarcophaginae in rural settlements in the Croatian part of Baranja and to emphasize the importance of these habitats for flesh fly diversity.

## ﻿Materials and methods

### ﻿Study area

**Figure 1.**
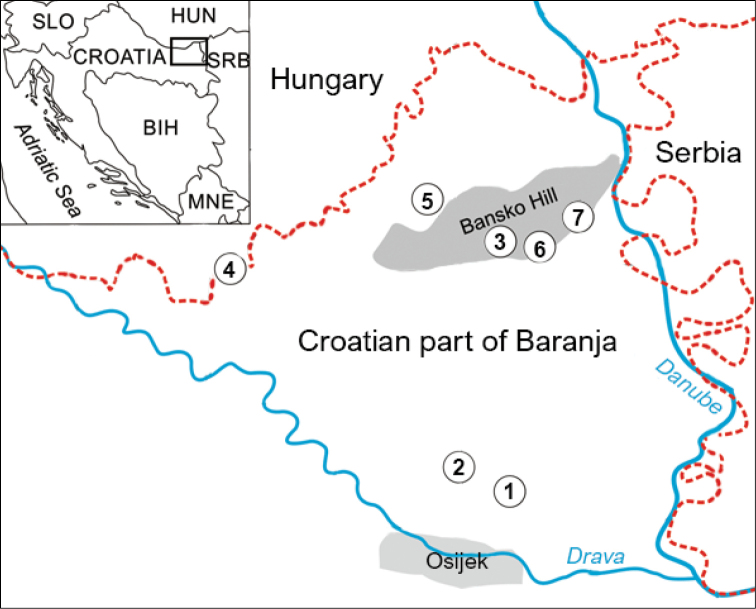
Sampling sites for flesh flies (Sarcophagidae: Sarcophaginae) in the Croatian part of Baranja. Legend: 1. Bilje; 2. Darda; 3. Kotlina; 4. Petlovac; 5. Popovac; 6. Suza; 7. Zmajevac.

Baranja is a Pannonian Plain region of Hungary (its northern portion) and Croatia (its southern portion). It is situated in the eastern part of Croatia and forms part of Osijek-Baranja County. Triangular in shape, it covers an area of 1147 km^2^ between the Drava, the Danube, and the state border with Hungary (Bognar et al. 1975). The Croatian part of Baranja is a predominantly lowland area (elevation ≤ 259 m). Bansko brdo (Bansko Hill) is the most prominent part of Baranja in terms of relief and extends NE-SW for 21 km, whereas its width is much smaller (Bognar et al. 1975). The steppe, the natural vegetation that covers Bansko Hill, has completely disappeared. The belt along the Danube and Drava is a flooded area (~ 63% of the territory) with many secondary tributaries and wetlands (Kopački rit) (Bognar et al. 1975). The Kopački rit Nature Park is one of the largest fluvial-marshy plains in Europe (Schneider-Jacoby 1994), and the basic ecological features relate to the river dynamics (Schneider-Jacoby 1994; Mihaljević et al. 1999). Forests cover ~ 20% of the Croatian part of Baranja. The climate is moderately continental with significant temperature fluctuations. The average January temperature is ~ -1.3 °C, and the July temperature is ~ 22 °C; the average annual rainfall is ~ 650 mm (Bognar et al. 1975). The Croatian part of Baranja contains 54 settlements, some of which contain pond habitats, which increasingly serve as places for recreation of people and their pets. All seven sampling sites are situated at the periphery of the settlements (Fig. 1). Geographical coordinates of these seven sampling sites are given in Table 1. Pond habitats in settlements of Kotlina, Suza, and Zmajevac are surrounded by species of willow (*Salix*) and poplar (*Populus*). Sampling sites in the settlements of Petlovac and Popovac are overgrown mainly with reeds (*Phragmitesaustralis*), sedges (*Carex* ssp.), and rush (*Typha* ssp.) without forest vegetation at its edges. A similar type of vegetation is present at the pond in Darda, with the addition of water lilies (*Nymphaeaalba*) and nenuphar (*Nupharluteum*), while pond habitats in Bilje settlement are overgrown with different species of low grasses exposed to the open sun throughout the day.

**Table 1.** List of sampling sites around pond habitats in Croatian part of Baranja.

No. of locality	Locality	Geographical coordinates
1	Bilje	45°36'16"N, 18°44'39"E
2	Darda	45°37'30"N, 18°41'21"E
3	Kotlina	45°47'17"N, 18°44'16"E
4	Petlovac	45°45'31"N, 18°31'41"E
5	Popovac	45°48'22"N, 18°39'34"E
6	Suza	45°46'55"N, 18°46'32"E
7	Zmajevac	45°48'03"N, 18°48'29"E

### ﻿Sampling and identification

Collections of sarcophagids from pond habitats were made frequently over a period of seven months (April–October) from 2019 to 2021. During 2019 and 2020, from April to October, sampling at the pond habitat in Zmajevac was done 1–5 times a month. Samplings in 2021 were carried out once per month from May to August. Flesh flies were sampled from 1 pm to 5 pm using a standard insect sweep net to selectively collect flies resting on soil and vegetation, or attracted to animal faeces and the remains of discarded food. The collected specimens were preserved in 96% ethanol. Male terminalia were prepared for species identification following the method of Richet et. al (2011). After two days in ethanol, male abdomens were dissected and soaked in a 10% KOH solution for 72 h. They were then immersed in 10% acetic acid for 1 min and rinsed with water for 1 min. They were then dehydrated in beech-wood creosote for 4 h. The phallus, pregonites and postgonites, sternite 5, cerci, and surstyli were separated from the rest of abdomen and placed into a plastic vial (volume of 2 ml) with 96% of ethanol solution. Identifications were carried out using keys for Sarcophagidae (Pape 1987; Povolný and Verves 1997; Richet et al. 2011) and descriptions and illustrations in Whitmore (2009, 2010, 2011) and Whitmore et al. (2013). Nomenclature and classification follow the Fauna Europaea database (Pape 2004). For all samples, the following information is provided: locality and date of collection, collector(s), number and sex of specimens, and depository. Also, 33 unidentified specimens from May 2018 are now identified and their data have been included in this study. One species newly recorded for the Croatian fauna is marked with a black triangle (▲). Specimens examined for this study are deposited in the collections of the Department of Biology, Josip Juraj Strossmayer University of Osijek, Osijek, Croatia (**DBUO**) and the Staatliches Museum für Naturkunde, Stuttgart, Germany (**SMNS**).

### ﻿Terminology

Subgeneric names are abbreviated as follows:

***Bel.*** *Bellieriomima*

***Ber.*** *Bercaea*

***Hel.*** *Helicophagella*

***Het.*** *Heteronychia*

***Kra.*** *Krameromyia*

***Lip.*** *Liopygia*

***Lis.*** *Liosarcophaga*

***Meh.*** *Mehria*

***Myo.*** *Myorhina*

***Pan.*** *Pandelleana*

***Pas.*** *Parasarcophaga*

***Pad.*** *Pandelleisca*

***Pse.*** *Pseudothyrsocnema*

***Rob.*** *Robineauella*

***Ros.*** *Rosellea*

***S.r.*** *Sarcophaga*

***S.c.*** *Sarcotachinella*

***Ser.*** *Servaisia*

***Thy.*** *Thyrsocnema*

## ﻿Results

A total of 1293 flesh flies belonging to 37 species was collected (Table 2). Sarcophaga (Sar.) croatica Baranov, 1941 was the most abundant with 37%, followed by S. (Sar.) lehmanni Müller, 1922 (21%), S. (Pas.) albiceps Meigen, 1826 (5%), S. (Sar.) baranoffi Rohdendorf, 1937 (5%), S. (Thy.) incisilobata Pandellé, 1896 (5%), S. (Lis.) emdeni (Rohdendorf, 1969) (3%), S. (Lis.) aegyptica Salem, 1935 (3%), S. (Hel.) melanura Meigen, 1826 (3%), S. (Het.) filia Rondani, 1860 (3%), S. (Ros.) aratrix Pandellé, 1896 (2%), S. (Hel.) noverca Rondani, 1860 (2%), S. (Bel.) subulata Pandellé, 1896 (1%), S. (Pad.) similis Meade, 1876 (1%), and *Raviniapernix* (Harris, 1780) (1%). These fourteen species comprised 92% of the samples; the remaining 23 species represented 8% (Table 2). Most species (35) and specimens (75%) were collected in Zmajevac (Table 2). The lowest number of species was collected in Bilje (3), whereas in other localities the number of collected species was between 8 and 11. The largest number of specimens and species was collected during 2019 (Table 3). New records for Baranja are provided for 25 species, with the record of S. (Pse.) spinosa representing a first record for Croatia.

**Table 2.** Number of species and specimens of flesh flies from subfamily Sarcophaginae collected at seven localities in Croatian part of Baranja from 2018 to 2021.

Species / Locality	1 Bilje	2 Darda	3 Kotlina	4 Petlovac	5 Popovac	6 Suza	7 Zmajevac	∑
Sarcophaga (Sar.) croatica	20	32	17	41	1	33	328	472
S. (Sar.) lehmanni	4	-	20	8	11	42	191	276
S. (Pas.) albiceps	6	-	1	-	-	-	59	66
S. (Sar.) baranoffi	-	2	2	2	-	4	52	62
S. (Thy.) incisilobata	-	1	5	5	1	3	44	59
S. (Lis.) emdeni	-	-	-	-	-	10	33	43
S. (Lis.) aegyptica	-	1	1	6	3	1	29	41
S. (Hel.) melanura	-	1	-	1	3	-	33	38
S. (Het.) filia	-	-	-	3	-	2	32	37
S. (Ros.) aratrix	-	1	-	-	1	5	25	32
S. (Hel.) noverca	-	-	-	-	-	-	29	29
S. (Bel.) subulata	-	-	-	-	-	-	18	18
S. (Pad.) similis	-	1	-	2	1	-	13	17
*Raviniapernix*	-	1	2	-	6	-	6	15
S. (Myo.) soror	-	-	-	-	-	-	12	12
S. (Het.) haemorrhoa	-	-	-	-	-	-	10	10
S. (Myo.) nigriventris	-	-	-	-	-	-	7	7
S. (Sar.) variegata	-	-	-	4	-	-	3	7
S. (Hel.) crassimargo	-	-	-	-	-	-	6	6
S. (Het.) pseudobenaci	-	-	-	2	-	-	4	6
S. (Het.) schineri	-	-	-	-	-	-	5	5
S. (Sar.) carnaria	-	-	3	-	-	-	2	5
S. (Lip.) argyrostoma	-	-	-	-	1	-	3	4
S. (Rob.) caerulescens	-	-	-	-	-	-	4	4
S. (Het.) proxima	-	-	-	-	-	-	3	3
S. (Het.) vagans	-	-	-	-	-	-	3	3
S. (Ber.) africa	-	-	-	-	-	-	2	2
S. (Hel.) hirticrus	-	-	-	-	-	-	2	2
S. (Het.) haemorrhoides	-	-	-	-	-	-	2	2
S. (Het.) pumila	-	-	-	-	1	-	1	2
S. (Lis.) tuberosa	-	-	-	-	-	1	1	2
S. (Het.) depressifrons	-	-	-	-	-	-	1	1
S. (Lip.) crassipalpis	-	-	-	-	1	-	-	1
S. (Lis.) dux	-	1	-	-	-	-	-	1
S. (Meh.) sexpunctata	-	-	-	-	-	-	1	1
S. (Pan.) protuberans	-	-	-	-	-	-	1	1
S. (Pse.) spinosa	-	-	-	-	-	-	1	1
∑ = 37	30	41	51	74	30	101	966	1293

### ﻿List of recorded species

#### ﻿Subfamily Sarcophaginae Macquart, 1834

**1. *Raviniapernix* (Harris, 1780)**

**New records for Croatian Baranja.** Zmajevac, 5.IX.2019, S. Krčmar leg. (1♂) (SMNS); same locality, 28.VI.2020, S. Krčmar leg. (2♂) (DBUO); same locality, 13.IX.2020, S. Krčmar leg. (2♂) (DBUO); same locality, 20.VI.2021, S. Krčmar leg. (1♂) (DBUO); Kotlina, 18.V.2021, S. Krčmar leg. (1♂) (DBUO); same locality, 18.VI.2021, S. Krčmar leg. (1♂) (DBUO); Popovac, 11.VI.2021, S. Krčmar leg. (1♂) (DBUO); same locality, 10.VII.2021, S. Krčmar leg. (5♂) (DBUO); Darda, 17.VI.2021, S. Krčmar leg. (1♂) (DBUO).

**2. Sarcophaga (Bellieriomima) subulata Pandellé, 1896**

**Records.** Zmajevac, 9.V.2018, S. Krčmar leg. (4♂) (DBUO); same locality, 22.IV.2019, S. Krčmar leg. (1♂) (DBUO); same locality, 29.VII.2019, S. Krčmar leg. (8♂) (DBUO); same locality, 8.V.2020, S. Krčmar leg. (1♂) (DBUO); same locality, 12.VI.2020, S. Krčmar leg. (1♂) (DBUO); same locality, 12.VIII.2020, S. Krčmar leg. (2♂) (DBUO); same locality, 11.V.2021, S. Krčmar leg. (1♂) (DBUO).

**3. Sarcophaga (Bercaea) africa (Wiedemann, 1824)**

**Records.** Zmajevac, 16.V.2019, S. Krčmar leg. (1♂) (DBUO); same locality, 24.VIII.2019, S. Krčmar leg. (1♂) (DBUO).

**4. Sarcophaga (Helicophagella) crassimargo Pandellé, 1896**

**Records.** Zmajevac, 17.VI.2019, S. Krčmar leg. (1♂) (SMNS); same locality, 24.VIII.2019, S. Krčmar leg. (1♂) (SMNS); same locality, 28.VI.2020, S. Krčmar leg. (3♂) (DBUO); same locality, 28.VII.2020, S. Krčmar leg. (1♂) (DBUO).

**5. Sarcophaga (Helicophagella) hirticrus Pandellé, 1896**

**Records.** Zmajevac, 5.IX.2019, S. Krčmar leg. (1♂) (SMNS); same locality, 8.V.2020, S. Krčmar leg. (1♂) (DBUO).

**6. Sarcophaga (Helicophagella) melanura Meigen, 1826**

**Records.** Zmajevac, 9.V.2018, S. Krčmar leg. (2♂) (DBUO); same locality, 19.VIII.2019, S. Krčmar leg. (1♂) (DBUO); same locality, 22.VIII.2019, S. Krčmar leg. (3♂) (DBUO); same locality, 24.VIII.2019, S. Krčmar leg. (1♂) (DBUO); same locality, 5.IX.2019, S. Krčmar leg. (3♂) (DBUO); same locality, 6.IX.2019, S. Krčmar leg. (9♂) (DBUO, SMNS); same locality, 17.IX.2019, S. Krčmar leg. (1♂) (DBUO); same locality, 30.IX.2019, S. Krčmar leg. (2♂) (DBUO); same locality, 28.VI.2020, S. Krčmar leg. (1♂) (DBUO); same locality, 28.VII.2020, S. Krčmar leg. (1♂) (DBUO); same locality, 20.VI.2021, S. Krčmar leg. (9♂) (DBUO).

**New records for Croatian Baranja.** Popovac, 11.VI.2021, S. Krčmar leg. (1♂) (DBUO); same locality, 10.VII.2021, S. Krčmar leg. (2♂) (DBUO); Petlovac, 18.VI.2021, S. Krčmar leg. (1♂) (DBUO); Darda, 19.VIII.2021, S. Krčmar leg. (1♂) (DBUO).

**7. Sarcophaga (Helicophagella) noverca Rondani, 1860**

**Records.** Zmajevac, 9.V.2018, S. Krčmar leg. (6♂) (DBUO); same locality, 16.V.2019, S. Krčmar leg. (2♂) (DBUO); same locality, 22.V.2019, S. Krčmar leg. (12♂) (DBUO); same locality, 22.VIII.2019, S. Krčmar leg. (2♂) (DBUO); same locality, 5.IX.2019, S. Krčmar leg. (1♂) (DBUO); same locality, 6.IX.2019, S. Krčmar leg. (1♂) (DBUO); same locality, 13.IX.2019, S. Krčmar leg. (1♂) (SMNS); same locality, 8.V.2020, S. Krčmar leg. (1♂) (DBUO); same locality, 12.VIII.2020, S. Krčmar leg. (2♂) (DBUO); same locality, 13.IX.2020, S. Krčmar leg. (1♂) (DBUO).

**8. Sarcophaga (Heteronychia) depressifrons Zetterstedt, 1845**

**New records for Croatian Baranja.** Zmajevac, 12.VIII.2020, S. Krčmar leg. (1♂) (DBUO).

**9. Sarcophaga (Heteronychia) filia Rondani, 1860**

**New records for Croatian Baranja.** Zmajevac, 6.IX.2019, S. Krčmar leg. (1♂) (SMNS); same locality, 12.VI.2020, S. Krčmar leg. (6♂) (DBUO); same locality, 19.VI.2020, S. Krčmar leg. (1♂) (DBUO); same locality, 28.VI.2020, S. Krčmar leg. (17♂) (DBUO); same locality, 28.VII.2020, S. Krčmar leg. (6♂) (DBUO); same locality, 12.VIII.2020, S. Krčmar leg. (1♂) (DBUO); Petlovac, 18.V.2021, S. Krčmar leg. (1♂) (DBUO); same locality, 18.VI.2021, S. Krčmar leg. (2♂) (DBUO); Suza, 18.V.2021, S. Krčmar leg. (2♂) (DBUO).

**10. Sarcophaga (Heteronychia) haemorrhoa Meigen, 1826**

**Records.** Zmajevac, 9.V.2018, S. Krčmar leg. (1♂) (SMNS); same locality, 16.V.2019, S. Krčmar leg. (1♂) (SMNS); same locality, 29.VI.2019, S. Krčmar leg. (1♂) (DBUO), same locality, 5.IX.2019, S. Krčmar leg. (1♂) (SMNS); same locality, 12.VI.2020, S. Krčmar leg. (1♂) (DBUO); same locality, 28.VI.2020, S. Krčmar leg. (3♂) (DBUO); same locality, 28.VII.2020, S. Krčmar leg. (1♂) (DBUO); same locality, 12.VIII.2020, S. Krčmar leg. (1♂) (DBUO).

**11. Sarcophaga (Heteronychia) haemorrhoides Böttcher, 1913**

**New records for Croatian Baranja.** Zmajevac, 28.VI.2020, S. Krčmar leg. (1♂) (DBUO); same locality, 12.VIII.2020, S. Krčmar leg. (1♂) (DBUO).

**12. Sarcophaga (Heteronychia) proxima Rondani, 1860**

**Records.** Zmajevac, 22.V.2019, S. Krčmar leg. (1♂) (SMNS); same locality, 24.VIII.2019, S. Krčmar leg. (1♂) (SMNS); same locality, 30.VIII.2019, S. Krčmar leg. (1♂) (SMNS).

**13. Sarcophaga (Heteronychia) pseudobenaci (Baranov, 1942)**

**Records.** Zmajevac, 29.VI.2019, S. Krčmar leg. (1♂) (DBUO); same locality, 8.V.2020, S. Krčmar leg. (1♂) (DBUO); same locality, 13.IX.2020, S. Krčmar leg. (2♂) (DBUO).

**New records for Croatian Baranja.** Petlovac, 29.VIII.2021, S. Krčmar leg. (2♂) (DBUO).

**14. Sarcophaga (Heteronychia) pumila Meigen, 1826**

**New records for Croatian Baranja.** Popovac, 11.VI.2021, S. Krčmar leg. (1♂) (DBUO); Zmajevac, 30.VII.2021, S. Krčmar leg. (1♂) (DBUO).

**15. Sarcophaga (Heteronychia) schineri Bezzi, 1891**

**Records.** Zmajevac, 9.V. 2018, S. Krčmar leg. (1♂) (SMNS); same locality, 22.V.2019, S. Krčmar leg. (1♂) (SMNS); same locality, 12.VI.2020, S. Krčmar leg. (1♂) (DBUO); same locality, 28.VI.2020, S. Krčmar leg. (1♂) (DBUO); same locality, 12.VIII.2020, S. Krčmar leg. (1♂) (DBUO).

**16. Sarcophaga (Heteronychia) vagans Meigen, 1826**

**New records for Croatian Baranja.** Zmajevac, 19.VIII.2019, S. Krčmar leg. (1♂) (SMNS); same locality, 19.VI.2020, S. Krčmar leg. (1♂) (DBUO); same locality, 28.VI.2020, S. Krčmar leg. (1♂) (DBUO).

**17. Sarcophaga (Liopygia) argyrostoma (Robineau-Desvoidy, 1830)**

**Records.** Zmajevac, 29.VII.2019, S. Krčmar leg. (1♂) (DBUO); same locality, 22.VIII.2019, S. Krčmar leg. (1♂) (SMNS); same locality, 6.IX.2019, S. Krčmar leg. (1♂) (SMNS).

**New records for Croatian Baranja.** Popovac, 13.VIII.2021, S. Krčmar leg. (1♂) (DBUO).

**18. Sarcophaga (Liopygia) crassipalpis Macquart, 1839**

**New records for Croatian Baranja.** Popovac, 10.VII.2021, S. Krčmar leg. (1♂) (DBUO).

**19. Sarcophaga (Liosarcophaga) aegyptica Salem, 1935**

**Records.** Zmajevac, 22.VIII.2019, S. Krčmar leg. (1♂) (DBUO); same locality, 24.VIII.2019, S. Krčmar leg. (2♂) (DBUO); same locality, 30.VIII.2019, S. Krčmar leg. (2♂) (SMNS); same locality, 5.IX.2019, S. Krčmar leg. (5♂) (DBUO); same locality, 6.IX.2019, S. Krčmar leg. (2♂) (SMNS); same locality, 13.IX.2019, S. Krčmar leg. (1♂) (DBUO); same locality, 17.IX.2019, S. Krčmar leg. (1♂) (DBUO); same locality, 30.IX.2019, S. Krčmar leg. (9♂) (DBUO); same locality, 12.VI.2020, S. Krčmar leg. (1♂) (DBUO); same locality, 28.VI.2020, S. Krčmar leg. (1♂) (DBUO); same locality, 13.IX.2020, S. Krčmar leg. (2♂) (DBUO); same locality, 20.VI.2021, S. Krčmar leg. (2♂) (DBUO).

**New records for Croatian Baranja.** Kotlina, 18.V.2021, S. Krčmar leg. (1♂) (DBUO); Darda, 17.VI.2021, S. Krčmar leg. (1♂) (DBUO); Petlovac, 18.VI.2021, S. Krčmar leg. (5♂) (DBUO); same locality, 20.VII.2021, S. Krčmar leg. (1♂) (DBUO); Suza, 25.VI.2021, S. Krčmar leg. (1♂) (DBUO); Popovac, 10.VII.2021, S. Krčmar leg. (2♂) (DBUO); same locality, 13.VIII.2021, S. Krčmar leg. (1♂) (DBUO).

**20. Sarcophaga (Liosarcophaga) dux Thomson, 1869**

**New records for Croatian Baranja.** Darda, 17.VI. 2021, S. Krčmar leg. (1♂) (DBUO).

**21. Sarcophaga (Liosarcophaga) emdeni (Rohdendorf, 1969)**

**Records.** Zmajevac, 9.V.2018, S. Krčmar leg. (1♂) (DBUO); same locality, 16.V.2019, S. Krčmar leg. (4♂) (DBUO); same locality, 22.V.2019, S. Krčmar leg. (3♂) (DBUO); same locality, 17.VI.2019, S. Krčmar leg. (2♂) (DBUO); same locality, 6.VII.2019, S. Krčmar leg. (4♂) (DBUO); same locality, 29.VII.2019, S. Krčmar leg. (2♂) (DBUO); same locality, 22.VIII.2019, S. Krčmar leg. (1♂) (DBUO); same locality, 24.VIII.2019, S. Krčmar leg. (2♂) (DBUO); same locality, 30.VIII.2019, S. Krčmar leg. (2♂) (DBUO); same locality, 5.IX.2019, S. Krčmar leg. (1♂) (DBUO); same locality, 13.IX.2019, S. Krčmar leg. (1♂) (DBUO); same locality, 8.V.2020, S. Krčmar leg. (1♂) (DBUO); same locality, 12.VI.2020, S. Krčmar leg. (1♂) (DBUO); same locality, 19.VI.2020, S. Krčmar leg. (1♂) (DBUO); same locality, 28.VI.2020, S. Krčmar leg. (4♂) (DBUO); same locality, 13.IX.2020, S. Krčmar leg. (1♂) (DBUO); same locality, 11.V.2021, S. Krčmar leg. (2♂) (DBUO).

**New records for Croatian Baranja.** Suza, 18.V.2021, S. Krčmar leg. (1♂) (DBUO); same locality, 11.VII.2021, S. Krčmar leg. (7♂) (DBUO); same locality, 12.VIII.2021, S. Krčmar leg. (2♂) (DBUO).

**22. Sarcophaga (Liosarcophaga) tuberosa Pandellé, 1896**

**New records for Croatian Baranja.** Zmajevac, 17.VI.2019, S. Krčmar leg. (1♂) (DBUO); Suza, 18.V.2021, S. Krčmar leg. (1♂) (DBUO).

**23. Sarcophaga (Mehria) sexpunctata (Fabricius, 1805)**

**New records for Croatian Baranja.** Zmajevac, 12.VI.2020, S. Krčmar leg. (1♂) (DBUO).

**24. Sarcophaga (Myorhina) nigriventris Meigen, 1826**

**Records.** Zmajevac, 16.V.2019, S. Krčmar leg. (1♂) (SMNS); same locality, 22.V.2019, S. Krčmar leg. (1♂) (DBUO); same locality, 30.IX.2019, S. Krčmar leg. (1♂) (SMNS); same locality, 28.VI.2020, S. Krčmar leg. (2♂) (DBUO); same locality, 28.VII.2020, S. Krčmar leg. (1♂) (DBUO); same locality, 20.X.2020, S. Krčmar leg. (1♂) (DBUO).

**25. Sarcophaga (Myorhina) soror Rondani, 1860**

**Records.** Zmajevac, 9.V.2018, S. Krčmar leg. (1♂) (DBUO); same locality, 16.V.2019, S. Krčmar leg. (3♂) (SMNS); same locality, 22.V.2019, S. Krčmar leg. (3♂) (DBUO); same locality, 29.VII.2019, S. Krčmar leg. (2♂) (DBUO); same locality, 19.VI.2020, S. Krčmar leg. (3♂) (DBUO).

**26. Sarcophaga (Pandelleana) protuberans Pandellé, 1896**

**New records for Croatian Baranja.** Zmajevac, 28.VI.2020, S. Krčmar leg. (1♂) (DBUO).

**27. Sarcophaga (Pandelleisca) similis Meade, 1876**

**Records.** Zmajevac, 16.V.2019, S. Krčmar leg. (1♂) (DBUO); same locality, 22.V.2019, S. Krčmar leg. (3♂) (DBUO); same locality, 24.VIII.2019, S. Krčmar leg. (1♂) (DBUO); same locality, 30.VIII.2019, S. Krčmar leg. (2♂) (SMNS); same locality, 5.IX.2019, S. Krčmar leg. (1♂) (DBUO); same locality, 6.IX.2019, S. Krčmar leg. (2♂) (DBUO); same locality, 13.IX.2019, S. Krčmar leg. (1♂) (DBUO); same locality, 30.IX.2019, S. Krčmar leg. (1♂) (DBUO); same locality, 28.VI.2020, S. Krčmar leg. (1♂) (DBUO).

**New records for Croatian Baranja.** Popovac, 11.VI.2021, S. Krčmar leg. (1♂) (DBUO); Petlovac, 18.VI.2021, S. Krčmar leg. (1♂) (DBUO); same locality, 20.VII.2021, S. Krčmar leg. (1♂) (DBUO); Darda, 19.VIII.2021, S. Krčmar leg. (1♂) (DBUO).

**28. Sarcophaga (Parasarcophaga) albiceps Meigen, 1826**

**Records.** Zmajevac, 16.V.2019, S. Krčmar leg. (1♂) (DBUO); same locality, 22.V.2019, S. Krčmar leg. (3♂) (DBUO); same locality, 29.VII.2019, S. Krčmar leg. (5♂) (DBUO); same locality, 19.VIII.2019, S. Krčmar leg. (1♂) (DBUO); same locality, 22.VIII.2019, S. Krčmar leg. (5♂) (DBUO); same locality, 24.VIII.2019, S. Krčmar leg. (4♂) (DBUO); same locality, 5.IX.2019, S. Krčmar leg. (5♂) (DBUO); same locality, 6.IX.2019, S. Krčmar leg. (16♂) (DBUO, SMNS); same locality, 8.V.2020, S. Krčmar leg. (1♂) (DBUO); same locality, 19.VI.2020, S. Krčmar leg. (2♂) (DBUO); same locality, 28.VI.2020, S. Krčmar leg. (7♂) (DBUO); same locality, 28.VII.2020, S. Krčmar leg. (2♂) (DBUO); same locality, 20.VI.2021, S. Krčmar leg. (7♂) (DBUO).

**New records for Croatian Baranja.** Bilje, 24.VII.2021, S. Krčmar leg. (6♂) (DBUO); Kotlina, 12.VIII.2021, S. Krčmar leg. (1♂) (DBUO).

**29. Sarcophaga (Robineauella) caerulescens Zetterstedt, 1838**

**Records.** Zmajevac, 22.V.2019, S. Krčmar leg. (1♂) (SMNS); same locality, 17.VI.2019, S. Krčmar leg. (1♂) (DBUO); same locality, 8.V.2020, S. Krčmar leg. (1♂) (DBUO); same locality, 28.VII.2020, S. Krčmar leg. (1♂) (DBUO).

**30. Sarcophaga (Rosellea) aratrix Pandellé, 1896**

**Records.** Zmajevac, 9.V.2018, S. Krčmar leg. (1♂) (DBUO); same locality, 22.IV.2019, S. Krčmar leg. (1♂) (SMNS); same locality, 16.V.2019, S. Krčmar leg. (2♂) (DBUO); same locality, 22.V.2019, S. Krčmar leg. (2♂) (SMNS); same locality, 17.VI.2019, S. Krčmar leg. (1♂) (DBUO); same locality, 24.VIII.2019, S. Krčmar leg. (1♂) (DBUO); same locality, 30.VIII.2019, S. Krčmar leg. (5♂) (DBUO); same locality, 5.IX.2019, S. Krčmar leg. (2♂) (DBUO); same locality, 8.V.2020, S. Krčmar leg. (3♂) (DBUO); same locality, 12.VI.2020, S. Krčmar leg. (1♂) (DBUO); same locality, 13.IX.2020, S. Krčmar leg. (2♂) (DBUO); same locality, 20.VI.2021, S. Krčmar leg. (2♂) (DBUO); same locality, 30.VII.2021, S. Krčmar leg. (1♂) (DBUO); same locality, 12.VIII.2021, S. Krčmar leg. (1♂) (DBUO).

**New records for Croatian Baranja.** Suza, 18.V.2021, S. Krčmar leg. (3♂) (DBUO); same locality, 11.VII.2021, S. Krčmar leg. (1♂) (DBUO); Petlovac, 20.VII.2021, S. Krčmar leg. (1♂) (DBUO); Darda, 24.VII.2021, S. Krčmar leg. (1♂) (DBUO); Popovac, 13.VIII.2021, S. Krčmar leg. (1♂) (DBUO).

**31. Sarcophaga (Sarcophaga) baranoffi Rohdendorf, 1937**

**Records.** Zmajevac, 9.V.2018, S. Krčmar leg. (2♂) (DBUO); same locality, 16.V.2019, S. Krčmar leg. (8♂) (DBUO, SMNS); same locality, 22.V.2019, S. Krčmar leg. (4♂) (DBUO); same locality, 29.VII.2019, S. Krčmar leg. (7♂) (DBUO); same locality, 22.VIII.2019, S. Krčmar leg. (1♂) (DBUO); same locality, 24.VIII.2019, S. Krčmar leg. (2♂) (DBUO); same locality, 30.VIII.2019, S. Krčmar leg. (4♂) (DBUO); same locality, 5.IX.2019, S. Krčmar leg. (1♂) (DBUO); same locality, 6.IX.2019, S. Krčmar leg. (6♂) (DBUO); same locality, 30.IX.2019, S. Krčmar leg. (1♂) (DBUO); same locality, 19.VI.2020, S. Krčmar leg. (1♂) (DBUO); same locality, 28.VI.2020, S. Krčmar leg. (2♂) (DBUO); same locality, 12.VIII.2020, S. Krčmar leg. (1♂) (DBUO); same locality, 13.IX.2020, S. Krčmar leg. (1♂) (DBUO); same locality, 11.V.2021, S. Krčmar leg. (1♂) (DBUO); same locality, 20.VI.2021, S. Krčmar leg. (1♂) (DBUO); same locality, 30.VII.2021, S. Krčmar leg. (6♂) (DBUO); same locality, 12.VIII.2021, S. Krčmar leg. (3♂) (DBUO).

**New records for Croatian Baranja.** Kotlina, 18.V.2021, S. Krčmar leg. (2♂) (DBUO); Suza, 25.VI.2021, S. Krčmar leg. (2♂) (DBUO); same locality, 12.VIII.2021, S. Krčmar leg. (2♂) (DBUO); Darda, 24.VII.2021, S. Krčmar leg. (2♂) (DBUO); Petlovac, 29.VIII.2021, S. Krčmar leg. (2♂) (DBUO).

**32. Sarcophaga (Sarcophaga) carnaria (Linnaeus, 1758)**

**New records for Croatian Baranja.** Zmajevac, 13.IX.2019, S. Krčmar leg. (1♂) (DBUO); same locality, 20.VI.2021, S. Krčmar leg. (1♂) (DBUO); Kotlina, 18.V.2021, S. Krčmar leg. (3♂) (DBUO).

**33. Sarcophaga (Sarcophaga) croatica Baranov, 1941**

**Records.** Zmajevac, 9.V.2018, S. Krčmar leg. (14♂) (DBUO); same locality, 22.IV.2019, S. Krčmar leg. (17♂) (DBUO, SMNS); same locality, 16.V.2019, S. Krčmar leg. (20♂) (DBUO); same locality, 22.V.2019, S. Krčmar leg. (15♂) (DBUO); same locality, 17.VI.2019, S. Krčmar leg. (10♂) (DBUO); same locality, 6.VII.2019, S. Krčmar leg. (1♂) (DBUO); same locality, 29.VII.2019, S. Krčmar leg. (5♂) (DBUO); same locality, 19.VIII.2019, S. Krčmar leg. (19♂) (DBUO); same locality, 22.VIII.2019, S. Krčmar leg. (19♂) (DBUO); same locality, 24.VIII.2019, S. Krčmar leg. (29♂) (DBUO); same locality, 30.VIII.2019, S. Krčmar leg. (34♂) (DBUO); same locality, 5.IX.2019, S. Krčmar leg. (9♂) (DBUO); same locality, 13.IX.2019, S. Krčmar leg. (8♂) (DBUO); same locality, 17.IX.2019, S. Krčmar leg. (1♂) (DBUO); same locality, 30.IX.2019, S. Krčmar leg. (3♂) (DBUO); same locality, 8.V.2020, S. Krčmar leg. (12♂) (DBUO); same locality, 12.VI.2020, S. Krčmar leg. (8♂) (DBUO); same locality, 19.VI.2020, S. Krčmar leg. (29♂) (DBUO); same locality, 28.VI.2020, S. Krčmar leg. (8♂) (DBUO); same locality, 28.VII.2020, S. Krčmar leg. (2♂) (DBUO); same locality, 12.VIII.2020, S. Krčmar leg. (3♂) (DBUO); same locality, 13.IX.2020, S. Krčmar leg. (22♂) (DBUO); same locality, 11.X.2020, S. Krčmar leg. (1♂) (DBUO); same locality, 20.X.2020, S. Krčmar leg. (5♂) (DBUO); same locality, 11.V.2021, S. Krčmar leg. (8♂) (DBUO); same locality, 20.VI.2021, S. Krčmar leg. (17♂) (DBUO); same locality, 30.VII.2021, S. Krčmar leg. (4♂) (DBUO); same locality, 12.VIII.2021, S. Krčmar leg. (5♂) (DBUO).

**New records for Croatian Baranja.** Kotlina, 18.V.2021, S. Krčmar leg. (8♂) (DBUO); same locality, 18.VI.2021, S. Krčmar leg. (9♂) (DBUO); Petlovac, 18.V.2021, S. Krčmar leg. (9♂) (DBUO); same locality, 18.VI.2021, S. Krčmar leg. (22♂) (DBUO); same locality, 20.VII.2021, S. Krčmar leg. (10♂) (DBUO); Suza, 18.V.2021, S. Krčmar leg. (6♂) (DBUO); same locality, 25.VI.2021, S. Krčmar leg. (17♂) (DBUO); same locality, 11.VII.2021, S. Krčmar leg. (5♂) (DBUO); same locality, 12.VIII.2021, S. Krčmar leg. (5♂) (DBUO); Popovac, 11.VI.2021, S. Krčmar leg. (1♂) (DBUO); Bilje, 17.VI.2021, S. Krčmar leg. (17♂) (DBUO); same locality, 19.VIII.2021, S. Krčmar leg. (3♂) (DBUO); Darda, 17.VI.2021, S. Krčmar leg. (2♂) (DBUO); same locality, 24.VII.2021, S. Krčmar leg. (16♂) (DBUO); same locality, 19.VIII.2021, S. Krčmar leg. (14♂) (DBUO).

**34. Sarcophaga (Sarcophaga) lehmanni Müller, 1922**

**Records.** Zmajevac, 22.IV.2019, S. Krčmar leg. (4♂) (DBUO, SMNS); same locality, 16.V.2019, S. Krčmar leg. (10♂) (DBUO); same locality, 22.V.2019, S. Krčmar leg. (19♂) (DBUO); same locality, 17.VI.2019, S. Krčmar leg. (6♂) (DBUO); same locality, 6.VII.2019, S. Krčmar leg. (5♂) (DBUO); same locality, 29.VII.2019, S. Krčmar leg. (3♂) (DBUO); same locality, 22.VIII.2019, S. Krčmar leg. (1♂) (DBUO); same locality, 24.VIII.2019, S. Krčmar leg. (3♂) (DBUO); same locality, 30.VIII.2019, S. Krčmar leg. (4♂) (DBUO); same locality, 5.IX.2019, S. Krčmar leg. (3♂) (DBUO); same locality, 6.IX.2019, S. Krčmar leg. (10♂) (DBUO); same locality, 13.IX.2019, S. Krčmar leg. (7♂) (DBUO); same locality, 17.IX.2019, S. Krčmar leg. (2♂) (DBUO); same locality, 30.IX.2019, S. Krčmar leg. (13♂) (DBUO); same locality, 29.VI.2019, S. Krčmar leg. (1♂) (DBUO); same locality, 8.V.2020, S. Krčmar leg. (17♂) (DBUO); same locality, 12.VI.2020, S. Krčmar leg. (18♂) (DBUO); same locality, 19.VI.2020, S. Krčmar leg. (12♂) (DBUO); same locality, 28.VI.2020, S. Krčmar leg. (16♂) (DBUO); same locality, 28.VII.2020, S. Krčmar leg. (6♂) (DBUO); same locality, 13.IX.2020, S. Krčmar leg. (6♂) (DBUO); same locality, 11.X.2020, S. Krčmar leg. (2♂) (DBUO); same locality, 20.X.2020, S. Krčmar leg. (4♂) (DBUO); same locality, 11.V.2021, S. Krčmar leg. (13♂) (DBUO); same locality, 20.VI.2021, S. Krčmar leg. (4♂) (DBUO); same locality, 12.VIII.2021, S. Krčmar leg. (2♂) (DBUO).

**New records for Croatian Baranja.** Kotlina, 18.V.2021, S. Krčmar leg. (9♂) (DBUO); same locality, 18.VI.2021, S. Krčmar leg. (10♂) (DBUO); same locality, 12.VIII.2021, S. Krčmar leg. (1♂) (DBUO); Petlovac, 18.V.2021, S. Krčmar leg. (3♂) (DBUO); same locality, 18.VI.2021, S. Krčmar leg. (3♂) (DBUO); same locality, 20.VII.2021, S. Krčmar leg. (2♂) (DBUO); Suza, 18.V.2021, S. Krčmar leg. (4♂) (DBUO); same locality, 25.VI.2021, S. Krčmar leg. (2♂) (DBUO); same locality, 11.VII.2021, S. Krčmar leg. (29♂) (DBUO); same locality, 12.VIII.2021, S. Krčmar leg. (7♂) (DBUO); Popovac, 11.VI.2021, S. Krčmar leg. (9♂) (DBUO); same locality, 13.VIII.2021, S. Krčmar leg. (2♂) (DBUO); Bilje, 17.VI.2021, S. Krčmar leg. (4♂) (DBUO).

**35. Sarcophaga (Sarcophaga) variegata (Scopoli, 1763)**

**New records for Croatian Baranja.** Zmajevac, 30.VIII.2019, S. Krčmar leg. (2♂) (SMNS); same locality, 11.V.2021, S. Krčmar leg. (1♂) (DBUO); Petlovac, 18.VI.2021, S. Krčmar leg. (4♂) (DBUO).

**36. Sarcophaga (Thyrsocnema) incisilobata Pandellé, 1896**

**Records.** Zmajevac, 16.V.2019, S. Krčmar leg. (2♂) (DBUO, SMNS); same locality, 17.VI.2019, S. Krčmar leg. (3♂) (DBUO); 22.VIII.2019, S. Krčmar leg. (1♂) (DBUO); same locality, 24.VIII.2019, S. Krčmar leg. (3♂) (DBUO); same locality, 5.IX.2019, S. Krčmar leg. (4♂) (DBUO); same locality, 6.IX.2019, S. Krčmar leg. (10♂) (DBUO); same locality, 8.V.2020, S. Krčmar leg. (2♂) (DBUO); same locality, 12.VI.2020, S. Krčmar leg. (1♂) (DBUO); same locality, 19.VI.2020, S. Krčmar leg. (2♂) (DBUO); same locality, 28.VI.2020, S. Krčmar leg. (3♂) (DBUO); same locality, 12.VIII.2020, S. Krčmar leg. (3♂) (DBUO); same locality, 11.X.2020, S. Krčmar leg. (1♂) (DBUO); same locality, 20.X.2020, S. Krčmar leg. (4♂) (DBUO); same locality, 20.VI.2021, S. Krčmar leg. (4♂) (DBUO); same locality, 12.VIII.2021, S. Krčmar leg. (1♂) (DBUO).

**New records for Croatian Baranja.** Petlovac, 18.V.2021, S. Krčmar leg. (3♂) (DBUO); same locality, 18.VI.2021, S. Krčmar leg. (1♂) (DBUO); same locality, 20.VII.2021, S. Krčmar leg. (1♂) (DBUO); Suza, 18.V.2021, S. Krčmar leg. (1♂) (DBUO); same locality, 25.VI.2021, S. Krčmar leg. (1♂) (DBUO); same locality, 11.VII.2021, S. Krčmar leg. (1♂) (DBUO); Darda, 17.VI.2021, S. Krčmar leg. (1♂) (DBUO); Kotlina, 18.VI.2021, S. Krčmar leg. (1♂) (DBUO); same locality, 20.VII.2021, S. Krčmar leg. (1♂) (DBUO); same locality, 12.VIII.2021, S. Krčmar leg. (3♂) (DBUO); Popovac, 13.VIII.2021, S. Krčmar leg. (1♂) (DBUO).

**37. Sarcophaga (Pseudothyrsocnema) spinosa Villeneuve, 1912** ▲

**New records for Croatian Baranja.** Zmajevac, 12.VIII.2020, S. Krčmar leg. (1♂) (SMNS). New for Croatia.

## ﻿Discussion

The first samples of flesh flies from Croatian Baranja were collected from 2014 to 2017 in the localities Zmajevac and Kamenac, at which time 29 species were recorded (Krčmar et al. 2019). Of those 29, the following five species were not confirmed in the present study: Blaesoxipha (S.r.) rossica Villeneuve, 1912; S. (Het.) benaci Böttcher, 1913; S. (Kra.) anaces Walker, 1849; S. (Lis.) portschinskyi (Rohdendorf, 1937), and S. (S.c.) sinuata Meigen, 1826. Meanwhile, 13 species were newly recorded for the area in this study. Of all the 42 species recorded from Croatian Baranja, 41 are widely distributed in Central Europe (Povolný and Verves 1997), whereas one, S. (Het.) pseudobenaci, is restricted to southeastern Europe (Pape 2004). Sarcophaga (Pse.) spinosa represents a new record for Croatia, which is not surprising as this species is recorded from neighbouring Hungary and Serbia, and has also been recorded from Albania, French mainland, Italian mainland, North Macedonia, Romania, and Ukraine (Pape 2004). Most species were found in natural and semi-natural habitats, although some of the species recorded in this study, i.e., S. (Ber.) africa, S. (Hel.) hirticrus, S. (Het.) depressifrons, S. (Lip.) argyrostoma, S. (Ros.) aratrix, and S. (Sar.) carnaria are known to be found also in urban habitats (Hwang and Turner 2005). Some species of significance for forensic entomology were collected on several localities around pond habitats in rural settlements, in particular S. (Lip.) argyrostoma and S. (Lis.) dux, both of which colonize decomposing human remains (Draber-Mońko et al. 2009; Sukontason et al. 2014), whereas S. (Lis.) dux has a medical importance as an agent of myiasis (Sukontason et al. 2014). Also, an important role of S. (Lip.) argyrostoma in accidental intestinal myiasis was recently confirmed (Najjari et al. 2020). Moreover, S. (Ber.) africa, S. (Lip.) argyrostoma, S. (Pad.) similis, and S. (Rob.) caerulescens have been found on corpses in indoor cases in Switzerland and Finland (Cherix et al. 2012; Ren et al. 2018), while S. (Lip.) crassipalpis was found on corpses at the earliest stage of decomposition in Australia (Ren et al. 2018). All these five species were recorded in this study (Table 2). Sarcophaga (Pad.) similis was recorded in four localities (Darda, Petlovac, Popovac, Zmajevac). Sarcophaga (Lip.) argyrostoma was recorded on two localities (Popovac, Zmajevac), S. (Ber.) africa, and S. (Rob.) caerulescens were only recorded in Zmajevac, while S. (Lip.) crassipalpis was recorded only in Popovac (Table 2). In this study, a large number of flesh fly specimens were collected on or nearby pet animal faeces and on discarded leftover food. This is not surprising since it is known that S. (Pas.) albiceps, S. (Thy.) incisilobata, and *Raviniapernix* visit excrements from humans and (other) animals (Papp 1992a, 1992b). Several species of flesh flies are also known to visit different animal carcasses (Szpila et al. 2015). Among them, the following species were recorded during this study: *R.pernix*, S. (Hel.) melanura, S. (Lip.) argyrostoma, S. (Pad.) similis, S. (Pas.) albiceps, S. (Rob.) caerulescens, S. (Ros.) aratrix, S. (Sar.) carnaria, S. (Sar.) lehmanni, S. (Sar.) variegata, and S. (Thy.) incisilobata. During this study not a single specimen of flesh flies was collected from carcasses. In Yozgat province of Turkey, 21 flesh flies species were recorded visiting small carrion (Pekbey 2019) from which S. (Ber.) africa, S. (Hel.) hirticrus, S. (Hel.) melanura, S. (Lip.) argyrostoma, S. (Lip.) crassipalpis, S. (Lis.) aegyptica, S. (Lis.) emdeni, S. (Lis.) tuberosa, S. (Myo.) nigriventris, S. (Sar.) lehmanni and S. (Thy.) incisilobata were also collected in this study. Eighty years ago, Baranov (1940) confirmed the presence of five flesh fly species in a laystall in the village of Metajna on the Island of Pag, four of which were also recorded in this study. In a similar study from the Polish Baltic coast, a number of species were recorded from a marshy habitat (15) and a sandy habitat (24) (Kaczorowska 2009), while in this study 37 species were recorded from pond habitats. Regardless of the distance and different climatic conditions, 11 species are present in all three types of habitats: S. (Ber.) africa, S. (Hel.) crassimargo, S. (Hel.) melanura, S. (Het.) haemorrhoa, S. (Het.) vagans, S. (Myo.) nigriventris, S. (Rob.) caerulescens, S. (Ros.) aratrix, S. (Sar.) carnaria, S. (S.c.) sinuata, and S. (Thy.) incisilobata. In marshy and sandy habitats the most numerous species was S. (Sar.) carnaria (Kaczorowska 2009), while S. (Sar.) croatica was the most abundant around pond habitats in rural settlements. Martínez-Sánchez et al. (2000) recorded 12 species from a holm-oak pasture agroecosystem in Salamanca province (Spain), from which four species S. (Ber.) africa, S. (Hel.) melanura, S. (Lip.) crassipalpis, and S. (Sar.) lehmanni were also collected in this study. The large differences in the number of recorded species between Zmajevac and other localities may be explained by the much higher number of samples. This is clearly shown by the fact that in 2021 only 15 species were collected at the Zmajevac locality compared to the total number of 35 species that were sampled during all three years (2019–2021) at this locality. The lower number of recorded species at the Bilje locality was influenced by environmental factors such as open sun throughout the day (with afternoon temperatures ≥ 32 °C) and a lack of animal faeces and remains for food, which reduced the number of recorded species. The seven localities around pond habitats are polluted by different organic contaminations caused by various human activities, which can attract certain species for feeding and breeding.

**Table 3.** Number of collected flesh flies from subfamily Sarcophaginae according to sampling years in the Croatian part of Baranja.

Species / Year	2018	2019	2020	2021	∑
Sarcophaga (Sar.) croatica	14	190	90	178	472
S. (Sar.) lehmanni	-	91	81	104	276
S. (Pas.) albiceps	-	40	12	14	66
S. (Sar.) baranoffi	2	34	5	21	62
S. (Thy.) incisilobata	-	23	16	20	59
S. (Lis.) emdeni	1	22	8	12	43
S. (Lis.) aegyptica	-	23	4	14	41
S. (Hel.) melanura	2	20	2	14	38
S. (Het.) filia	-	1	31	5	37
S. (Ros.) aratrix	1	14	6	11	32
S. (Hel.) noverca	6	19	4	-	29
S. (Bel.) subulata	4	9	4	1	18
S. (Pad.) similis	-	12	1	4	17
*Raviniapernix*	-	1	4	10	15
S. (Myo.) soror	1	8	3	-	12
S. (Het.) haemorrhoa	1	3	6	-	10
S. (Myo.) nigriventris	-	3	4	-	7
S. (Sar.) variegata	-	2	-	5	7
S. (Hel.) crassimargo	-	2	4	-	6
S. (Het.) pseudobenaci	-	1	3	2	6
S. (Het.) schineri	1	1	3	-	5
S. (Sar.) carnaria	-	1	-	4	5
S. (Lip.) argyrostoma	-	3	-	1	4
S. (Rob.) caerulescens	-	2	2	-	4
S. (Het.) proxima	-	3	-	-	3
S. (Het.) vagans	-	1	2	-	3
S. (Ber.) africa	-	2	-	-	2
S. (Hel.) hirticrus	-	1	1	-	2
S. (Het.) haemorrhoides	-	-	2	-	2
S. (Het.) pumila	-	-	-	2	2
S. (Lis.) tuberosa	-	1	-	1	2
S. (Het.) depressifrons	-	-	1	-	1
S. (Lip.) crassipalpis	-	-	-	1	1
S. (Lis.) dux	-	-	-	1	1
S. (Meh.) sexpunctata	-	-	1	-	1
S. (Pan.) protuberans	-	-	1	-	1
S. (Pse.) spinosa	-	-	1	-	1
∑ = 37	33	533	302	425	1293

## ﻿Conclusions

The species S. (Sar.) croatica and S. (Sar.) lehmanni were recorded from almost all sampling sites around pond habitats in the Croatian part of Baranja. They belong to the most widespread and abundant flesh flies in Croatia and have been recorded in all three biogeographic regions: Alpine, Mediterranean, and Pannonian–Peripannonian. By merging data from earlier studies (2014–2017) and this study, altogether 42 species of flesh flies were recorded in the Croatian part of Baranja. The species S. (Pse.) spinosa is recorded as new for the Croatian fauna, thus increasing the total number of recorded species of flesh flies in Croatia to 156.
